# Synergistic Catalysis of SnO_2_-CNTs Composite for ${\text{VO}}_{2}^{+}$/VO^2+^ and V^2+^/V^3+^ Redox Reactions

**DOI:** 10.3389/fchem.2021.671575

**Published:** 2021-05-05

**Authors:** Xiaojian Feng, Jing Xue, Tongxue Zhang, Zixuan Zhang, Chao Han, Lei Dai, Ling Wang, Zhangxing He

**Affiliations:** School of Chemical Engineering, North China University of Science and Technology, Tangshan, China

**Keywords:** vanadium redox flow battery, electrocatalyst, tin dioxide, carbon nanotubes, energy storage

## Abstract

In this study, a SnO_2_-carbon nanotube (SnO_2_-CNT) composite as a catalyst for vanadium redox flow battery (VRFB) was prepared using a sol-gel method. The effects of this composite on the electrochemical performance of ${\text{VO}}_{2}^{+}$/VO^2+^, and on the V^2+^/V^3+^ redox reactions and VRFB performance were investigated. The SnO_2_-CNT composite has better catalytic activity than pure SnO_2_ and CNT due to the synergistic catalysis of SnO_2_ and the CNT. SnO_2_ mainly provides the catalytic active sites and the CNTs mainly provide the three-dimensional structure and high electrical conductivity. Therefore, the SnO_2_-CNT composite has a larger specific surface area and an excellent synergistic catalytic performance. For cell performance, it was found that the SnO_2_-CNT cell shows a greater discharge capacity and energy efficiency. In particular, at 150 mA cm^−2^, the discharge capacity of the SnO_2_-CNT cell is 28.6 mAh higher than that of the pristine cell. The energy efficiency of the modified cell (7%) is 7.2% higher than that of the pristine cell (62.8%). This study shows that the SnO_2_-CNT is an efficient and promising catalyst for VRFB.

## Introduction

With the rapid development of society, humanity is consuming increasingly more natural energy from finite resources, such as coal and natural gas (He G. et al., 2020; Wang T. et al., 2020; Wang Z.-Y. et al., 2020). Furthermore, environmental pollution and energy shortages are becoming exacerbated (Cheng C. et al., 2020; Chuanchang et al., 2020; Huang et al., 2020a; Yang et al., 2020). Therefore, new energy development technologies have received widespread attention (Huang et al., 2020b; Wang D. et al., 2020; Liu et al., 2021; Nie et al., 2021). However, new resources, such as solar and tidal energies, cannot provide a continuous source of energy. It is therefore necessary to develop a large-scale electric energy storage system to integrate renewable energy (Kou et al., 2020; Wang Z. et al., 2020). Vanadium redox flow batteries (VRFBs) have the merits of flexible design, short response time, and long cycle life, resulting in them becoming popular large-scale energy storage devices (Wu et al., 2016; Ye et al., 2020; Jiang et al., 2021a).

VRFBs are mainly composed of electrolytes, ion exchange membranes, and electrodes (Jiang et al., 2021b). The positive and negative electrolytes are composed of ${\text{VO}}_{2}^{+}$/VO^2+^ and V^2+^/V^3+^ solutions, respectively (Cheng D. et al., 2020). The proton exchange membrane prevents electrolyte cross-contamination and proton transfer. The electrode is where the electrochemical reaction takes place and is therefore an important part of a VRFB (Lv et al., 2021). Carbon-based materials [carbon paper, carbon felt, graphite felt (GF), and so on] are popularly used as electrode materials for VRFB due to their good conductivity, large specific surface area, and low cost. However, their poor electrochemical activity limits the potential improvement in electrode performance (Yu et al., 2019).

In recent years, researchers have made excellent progress in improving the electrochemical performance of electrodes, including the introduction of catalysts. Currently, catalysts primarily include metals, metal oxides, and carbon-based materials. Regarding metal catalysts [e.g., Cu (Wei et al., 2016), Ir (Wang et al., 2007) and Sb (Kou et al., 2020)], Zhou et al. (2020) used semi-embedded carbon felt with bismuth nanospheres, which had good catalytic activity and could effectively promote the redox reaction of the negative electrode. For metal oxides [e.g., Nd_2_O_3_ (Fetyan et al., 2018), MnO_2_ (Ma et al., 2018), and PbO_2_ (Wu et al., 2014)], Bayeh et al. (2020) modified GF with cubic CeO_2_ nanowires and used it as a catalyst for VRFB. This resulted in abundant defects on the electrode surface and increased active sites, meaning that the CeO_2_ nanowires could significantly improve the ${\text{VO}}_{2}^{+}$/VO^2+^ redox process.

Regarding carbon-based materials [e.g., carbon nanosheets (He Z. et al., 2020), carbon nanofibers (Jiang et al., 2020), and graphene (Etesami et al., 2018)], Park et al. (2013) demonstrated that carbon nanofiber/nanotube composite catalysts had good electrocatalytic performance in VRFB. Compared with the untreated electrode, the discharge capacity of the modified electrode increased by 64% at 40 mA cm^−2^. This excellent performance is attributed to the increase in electron transfer rate by the modification treatment. The methods mentioned earlier accelerated the kinetics of redox reactions.

SnO_2_ is an amphoteric oxide with wide bandgap semiconductor properties and has been widely used in the field of electrocatalysis (Liu et al., 2016; Zhang et al., 2020). For example, Qiu et al. (2020) introduced SnO_2_ nanoparticles into carbon foam through an electrodeposition method as an anode for K-ion battery. This promoted electrolyte permeation and the transfer of K ions, resulting in an excellent cycle stability of the modified cell. Also, Mehboob et al. (2018) reported the electrocatalytic effect of SnO_2_ in VRFB. Carbon felt was modified with nano-SnO_2_ through a hydrothermal method. The stable and efficient performance of SnO_2_ was proven and it had excellent catalytic performance, although its conductivity was poor.

To solve this problem, a SnO_2_-carbon nanotube (SnO_2_-CNT) composite was prepared in this study using a sol-gel method. This composite had better catalytic activity than SnO_2_ and CNT separately due to its synergistic advantages. SnO_2_ primarily provided the catalytic active sites while the CNT provided the three-dimensional (3D) structure and high electrical conductivity. As a result, this SnO_2_-CNT composite had a large specific surface area and excellent synergistic catalytic properties, thereby having an excellent electrocatalytic performance as a bifunctional catalyst in VRFBs.

## Experiment

### Preparation of Materials

H_2_SO_4_ (98% purity) was purchased from China Beijing Chemical Reagent Co., Ltd. (Beijing, China). N, N-dimethylformamide (DMF, 99.5% purity) was purchased from Tianjin Yongda Chemical Reagent Co., Ltd. (Tianjin, China). CNT (300 mg) was dipped in concentrated sulfuric acid at 80°C for 8 h. After washing and filtering until neutral, the CNTs were put in an oven to dry for 24 h and were ground for later use. For the preparation of the active CNT, SnCl_2_·2H_2_O was first added to 20 ml of anhydrous ethanol, stirred at 80°C for 2 h, and aged for 24 h. Finally, the precursor of the tin sol was obtained. Activated CNT (SnCl_2_·2H_2_O and CNT at a molar mass of 1:13, respectively) and sol were mixed ultrasonically for 40 min and dried at 60°C. The mixture was heated at 500°C for 2 h in a nitrogen atmosphere in a tubular furnace. To obtain the SnO_2_-CNT composite, 149.7 mg of SnCl_2_·2H_2_O were added to 50 ml of anhydrous ethanol, stirred, and aged. Then, the precursor of tin sol was prepared. After drying, it was put into a tubular furnace and heated with nitrogen. This process was used to obtain SnO_2_.

### Characterization

The crystallographic phase of the sample was studied using X-ray diffraction (XRD) using a D8 Advance A25 Instrument (Bruker, Berlin, Germany). The morphology of the material was investigated using a scanning electron microscopy (SEM, JSM-IT100, JEOL, Japan). An X-ray photoelectron spectroscopy (XPS) was carried out using a Thermo Scientific (Waltham, MA, USA) ESCALAB 250Xi (Xi+) instrument.

### Electrochemical Measurements

An electrochemical test was performed with an electrochemical workstation (CHI660E, Shanghai Chenhua Instrument Co., Ltd., Beijing, China) using a three-electrode system. The glassy carbon electrode was the working electrode. The counter and reference electrodes were Pt and saturated calomel electrodes, respectively. The 10 mg sample was added to 5 ml of DMF and then sonicated for 3 h to make it uniformly mixed. A volume of 20 μl of the dispersion was transferred to the syringe, with 1–2 μl of the dispersion was added each time to the working electrode. The electrode was then dried for 4 h at indoor temperature. Cyclic voltammetry (CV) and electrochemical impedance spectroscopy (EIS) tests were carried out in a 1.6 M VO^2+^ + 3 M H_2_SO_4_ solution for the positive reaction and a 1.6 M V^3+^ + 3 M H_2_SO_4_ solution for the negative reaction. The positive voltage range was.2 to 1.5 V and the negative voltage range was −0.8 to −0.1 V. EIS was conducted with a frequency range of 10^−1^ to 10^6^ Hz. Polarization voltages of 0.85 and −0.45 V were used for the positive and negative redox reactions, respectively.

### Charge–Discharge Tests

To compare the cells' performance, modified and pristine cells were assembled. Charge–discharge tests were carried out by the battery test system (CT2001A, Wuhan, China). The voltage window was 0.7–1.65 V. The SnO_2_-CNT was used to modify the negative and positive electrodes of the modified cell. Original GF was used for the negative and positive electrodes of the pristine cell. The two poles of the cell were separated by a membrane. To prepare the modified GF, 10 mg of the SnO_2_-CNT was added to 10 mL of DMF. The GF (3 × 3 cm^2^) was soaked in this solution, ultrasonically dispersed for 3 h, and then dried in an oven. The original and modified GFs were soaked in an electrolyte of 0.8 M V^3+^ + 0.8 M VO^2+^ + 3 M H_2_SO_4_ in advance to completely assimilate the electrolyte. To balance the electrolytes, the cell was first charge–discharge for three cycles at a current density of 10 mA cm^−2^. Five charge–discharge tests were then performed at each current density of 50, 75, 100, 125, and 150 mA cm^−2^.

## Results and Discussion

**Figure 1:**
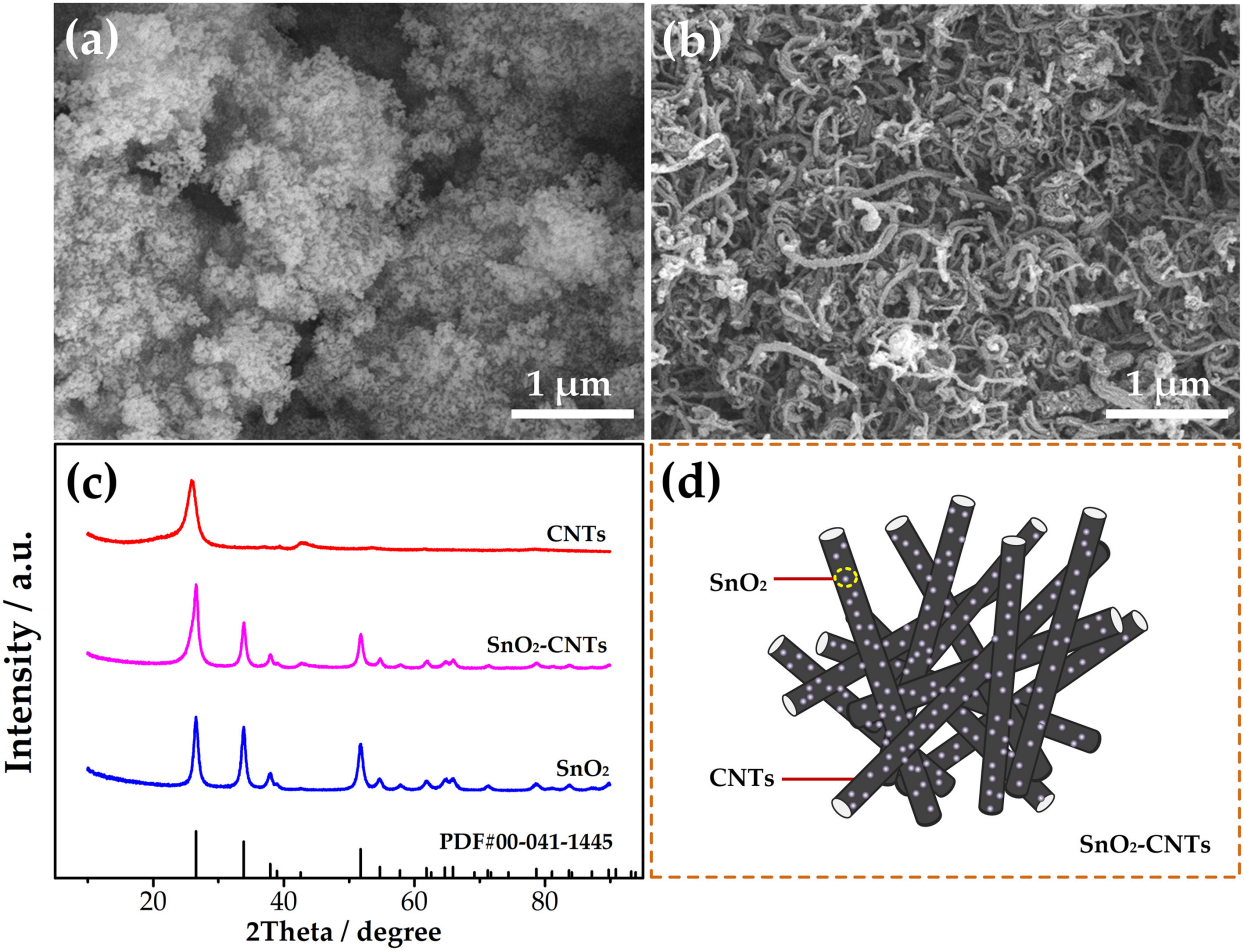
Scanning electron microscope images for **(a)** SnO_2_ and **(b)** SnO_2_-CNTs; **(c)** XRD for all samples; **(d)** structural diagram for SnO_2_-CNTs. SnO2-CNTs, SnO2-carbon nanotubes; XPS, X-ray photoelectron spectroscopy.

SEM characterization was used to research the morphology of SnO_2_ and SnO_2_-CNT. It can be seen from Figures 1a,b that the samples are all nanoscale. SnO_2_ exhibits a certain agglomeration but this is not obvious for the SnO_2_-CNT. XRD was used to study the crystal structure of the samples. As seen in Figure 1c, the CNTs have characteristic peaks at 26° and 42°, which prove that their purity is high. The peaks observed for SnO_2_ are consistent with the standard values (Nie et al., 2021) and there are no characteristic impurity peaks. The observed peaks of the SnO_2_-CNTs correspond with No. 00-041-1445. It can be seen that the peak of the composite material includes both pure CNTs and SnO_2_ peaks, indicating that the sample is a SnO_2_-CNTs composite material. The composite presents a long tubular morphology and SnO_2_ is attached to the CNTs, as shown in Figure 1d. Such a structure can provide a larger specific surface area, thus enhancing the catalytic performance. The composite combines the advantages of the two materials and greatly improves the electrochemical catalytic performance.

**Figure 2:**
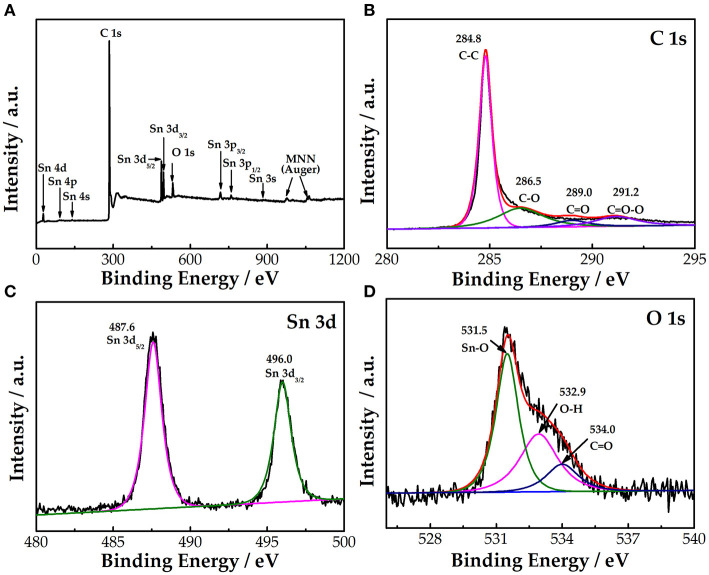
**(A)** XPS survey spectra of SnO_2_-CNTs; **(B)** high-resolution spectra for C1s; **(C)** Sn 3d; and **(D)** O 1s of SnO_2_-CNTs. SnO2-CNTs, SnO2-carbon nanotubes; XPS, X-ray photoelectron spectroscopy.

XPS was used to investigate the elemental composition of the SnO_2_-CNTs, as shown in Figure 2A. The sample has obvious peaks corresponding to C, Sn, and O, indicating that it mainly contains three elements. Figures 2B–D show the fitting results for each element. As seen in Figure 2B, the spectrum of C 1s is divided into four peaks at 284.8, 286.5, 289, and 291.2 eV, corresponding to the functional groups of C–C, C–O, C=O, and C=O–O, respectively (Tang et al., 2015). As seen in Figure 2C, Sn 3d_5/2_ and Sn 3d_3/2_ constitute the Sn 3d spectrum of the SnO_2_-CNTs. The peaks at 487.6 and 496 eV are assigned to Sn 3d_5/2_ and Sn 3d_3/2_, respectively (Tang et al., 2015). The splitting energy of the two peaks is 8.4 eV, indicating that the Sn element exists in the form of Sn^4+^ (Tian et al., 2020). Figure 2D displays the O 1s spectrum of the composite. The three peaks of the O 1s spectrum are assigned to the Sn–O (531.5 eV), O–H (532.9 eV), and C=O (534 eV) functional groups (Cheng et al., 2017).

**Figure 3:**
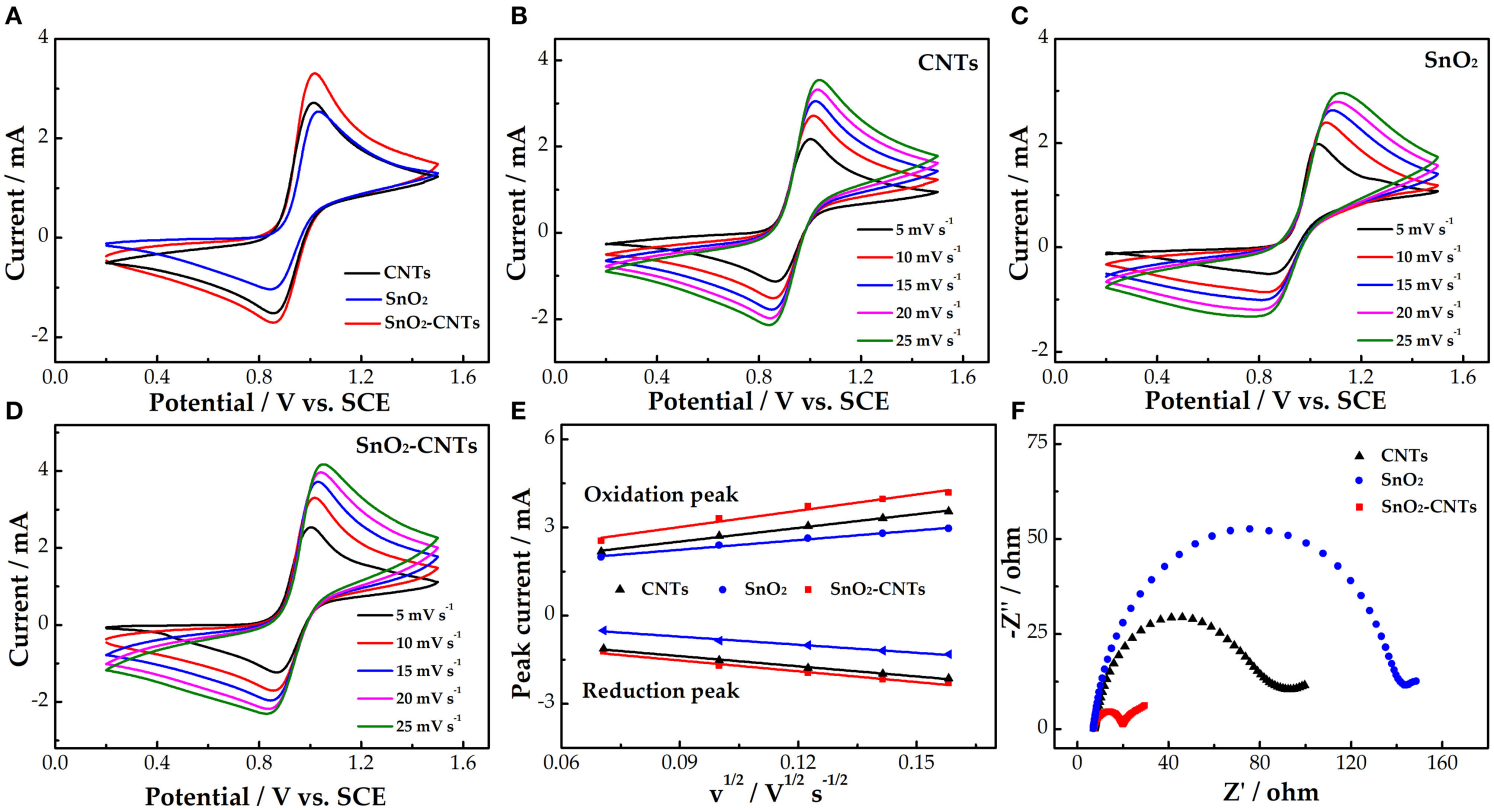
**(A)** CV curves for CNTs, SnO_2_, and SnO_2_-CNTs carried out in 1.6 M VO^2+^ + 3 M H_2_SO_4_ electrolyte of scan rate of 10 mV s^−1^; **(B)** CVs for CNTs; **(C)** SnO_2_; and **(D)** SnO_2_-CNTs at scan rates from 5 to 25 mV s^−1^ carried out in 1.6 M VO^2+^ + 3 M H_2_SO_4_ electrolyte; **(E)** plots of the redox peak current vs. the square root of the scan rate for SnO_2_, CNTs and SnO_2_-CNT electrodes; **(F)** positive Nyquist plots for SnO_2_, CNTs, and SnO_2_-CNTs carried out in 1.6 M VO^2+^ + 3.0 M H_2_SO_4_ electrolyte at 0.85 V. CV, cyclic voltammetry; SnO2-CNTs, SnO2-carbon nanotubes.

Figure 3A demonstrates the CV curves of the different electrodes. The order of electrochemical kinetics for the ${\text{VO}}_{2}^{+}$/VO^2+^ redox reaction is SnO_2_-CNTs > CNTs > SnO_2_. The redox peak current of the CNTs is higher than that of SnO_2_ due to the poor electrical conductivity of SnO_2_. The oxidation (3.3 mA) and reduction peak currents (1.7 mA) of the SnO_2_-CNTs both are the highest. This is due to the synergistic catalysis of SnO_2_ and the CNTs. SnO_2_ mainly provides the catalytic active sites while the CNTs provides the 3D structure and high electrical conductivity. The composite electrode shows the best electrocatalytic activity for the ${\text{VO}}_{2}^{+}$/VO^2+^ redox reaction.

Figures 3B–D show the CV curves of the CNTs, SnO_2_, and SnO_2_-CNTs, respectively. With increasing scan rate, the peak shape of the CV curves remains good, which proves that the electrodes have good electrochemical stability. Also, the redox peak current and peak potential difference increase with increasing scan rate. Figure 3E shows the redox peak current vs. the square root of the scan rate. The peak current is proportional to the square root of the scan rate for all samples, proving that the redox reaction is dominated by ion diffusion. The SnO_2_-CNTs have the highest linear slope. This may be due to the synergistic catalytic effect of composite material, which provides active sites for vanadium ions and promotes the mass transfer process of vanadium ions in solution.

Figure 3F shows the Nyquist diagrams for the three electrodes, each consisting of a semicircle of high frequency and a linear part of low frequency, corresponding to charge transfer and diffusion processes, respectively. A smaller semicircle diameter means a smaller charge transfer resistance. The charge transfer resistance of the SnO_2_-CNTs is much smaller than that of the other two samples. This may be due to the fact that the composite has a larger active surface area and more active sites than the CNT, which improves the catalytic performance of the vanadium redox reaction.

**Figure 4:**
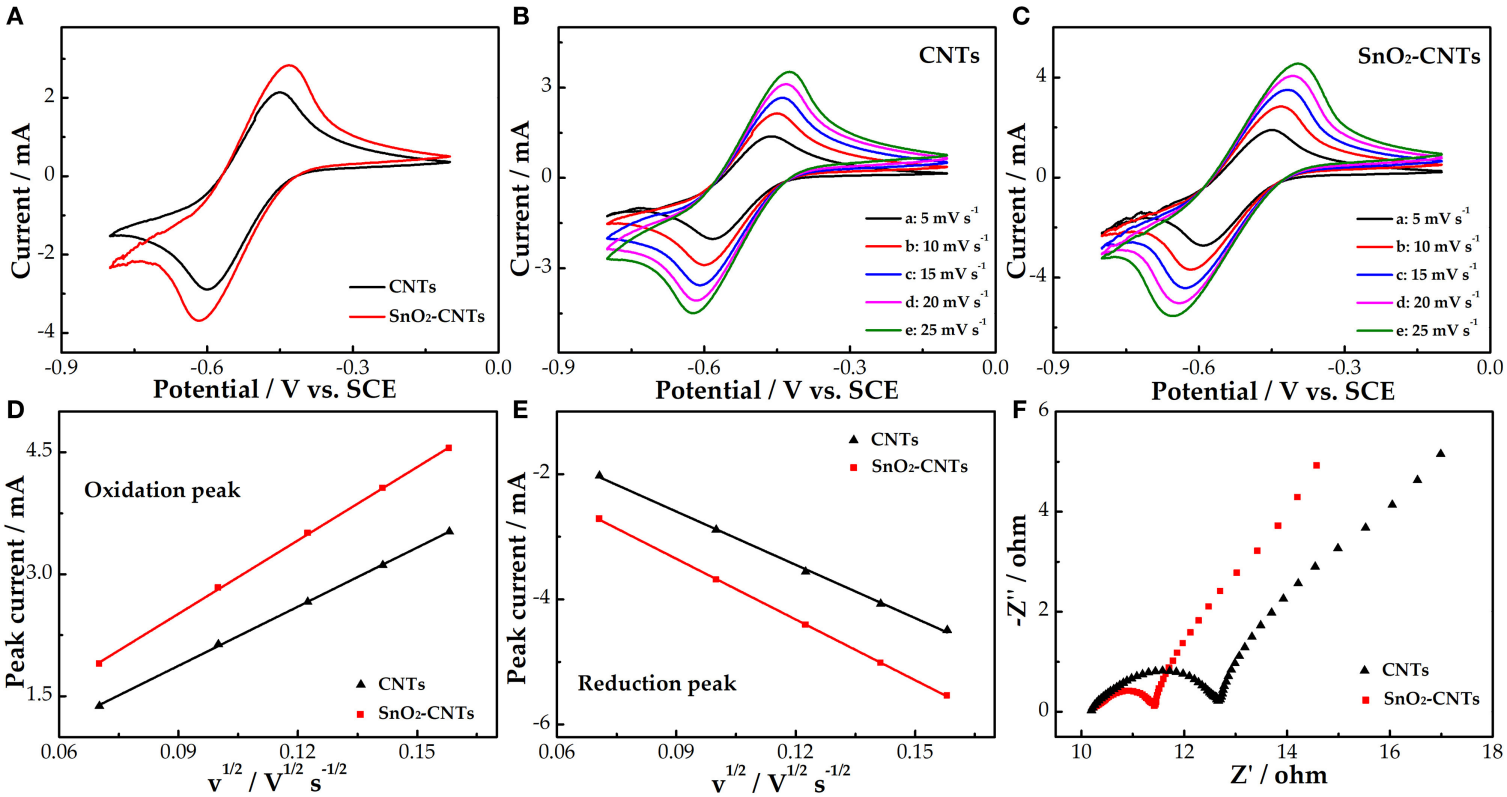
CV curves for CNTs and SnO_2_-CNTs carried out in 1.6 M V^3+^ + 3.0 M H_2_SO_4_ electrolyte at a scan rate of 10 mV s^−1^ **(A)**; CVs for CNTs **(B)** and SnO_2_-CNTs **(C)** at scan rates from 5 to 25 mV s^−1^ in the 1.6 M V^3+^ + 3 M H_2_SO_4_ electrolyte; plots of the redox peak current vs. the square root of the scan rate for CNTs and SnO_2_-CNT electrodes **(D,E)**; negative Nyquist plots for CNTs and SnO_2_-CNTs carried out in 1.6 M V^3+^ + 3 M H_2_SO_4_ electrolyte at a polarization potential of −0.45 V **(F)**. CV, cyclic voltammetry; SnO_2_-CNTs, SnO_2_-carbon nanotubes.

Figure 4A shows the negative CV curves of the CNTs and SnO_2_-CNTs. Both electrodes have obvious redox peaks and the peak redox current of the composite is larger than that of the CNTs, indicating that the SnO_2_-CNT electrode also shows better electrocatalytic activity for the V^2+^/V^3+^ redox reaction. Figures 4B,C show a series of CV curves for the CNTs and SnO_2_-CNTs composite, respectively, at different scan rates. The shape of the redox peak of the two electrodes remains unchanged. Under the influence of electrochemical polarization, the redox peak potential difference of the two electrodes increases with increasing scan rate.

The curves of the redox peak current vs. the square root of the scan rate can be seen in Figures 4D,E. The redox peak current is proportional to the square root of the scan rate. This shows that the V^2+^/V^3+^ redox reaction is controlled by a mass transfer process. The higher the linear slope, the higher the mass transfer rate. The linear slope of the SnO_2_-CNTs is higher than the CNTs. This may be due to the fact that the addition of SnO_2_ endows the CNTs with higher catalytic activity and more active sites. Figure 4F shows that the Nyquist diagrams of both the CNTs and SnO_2_-CNTs are composed of a semicircle of a high-frequency part and an oblique line of a low-frequency part. They correspond to the charge transfer and diffusion processes, respectively. The charge transfer resistance of the composite is lower, which indicates that it has a larger active surface area and more active sites than the CNTs, thus improving the kinetics of the vanadium redox reaction.

**Figure 5:**
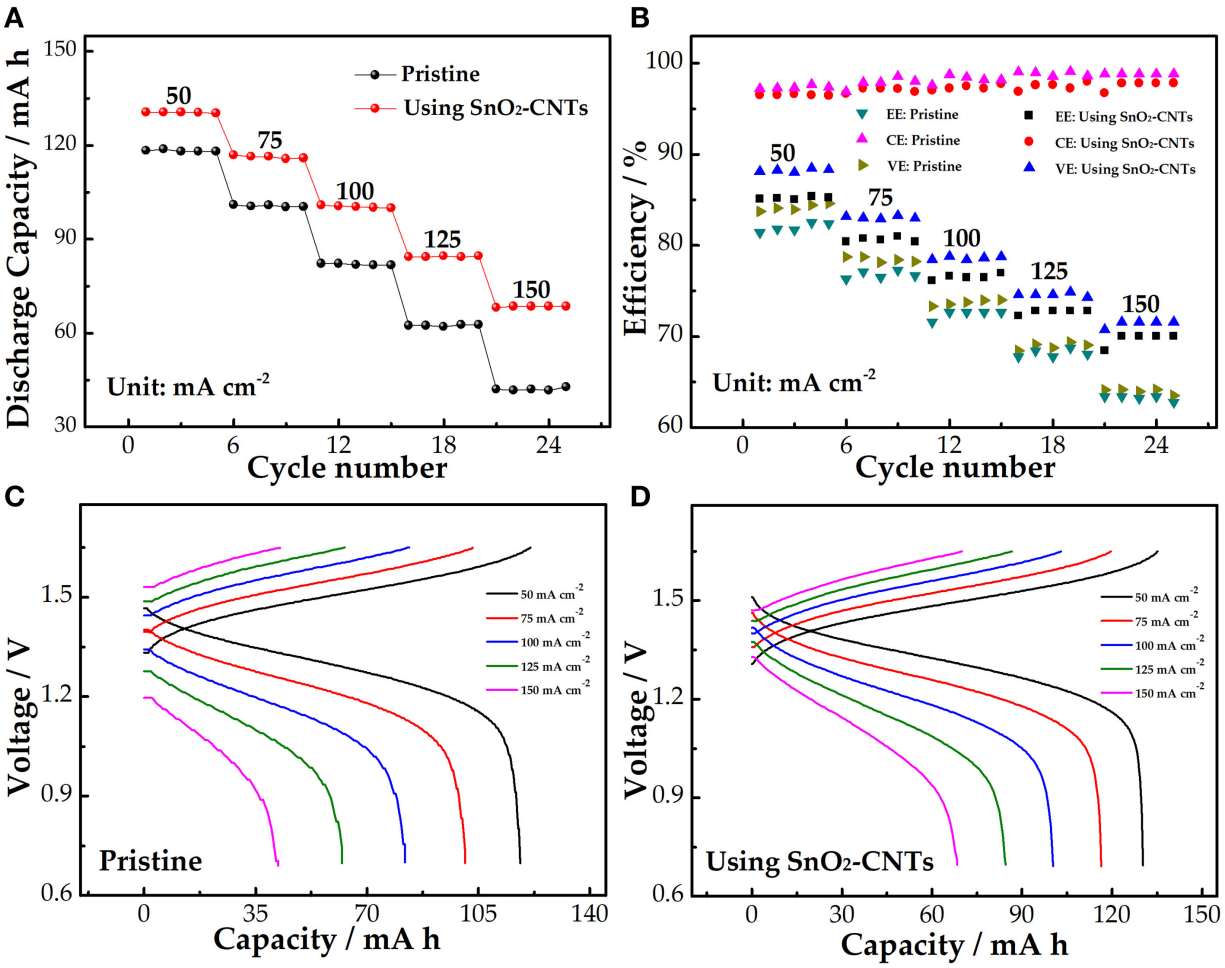
**(A)** Discharge capacity; **(B)** efficiency; **(C)** charge–discharge curves of the pristine cell **(C)**; and **(D)** SnO_2_-CNTs-modified cell at a current density of 50–150 mA cm^−2^. SnO_2_-CNTs, SnO_2_-carbon nanotubes.

The comparison of the charge and discharge rate performance of the pristine cell and the cell using the SnO_2_-CNTs was studied. As shown in Figure 5A, the discharge capacity gradually decreases because of the more severe electrochemical polarization at the high current density. The discharge capacity of the SnO_2_-CNT cell is 28.6 mAh higher than the pristine cell at 150 mA cm^−2^, indicating that the composite improves the utilization rate of the electrolyte.

Figure 5B shows the efficiency of the pristine and modified cells. The coulombic efficiency (CE) represents the ratio of the discharge capacity to charge capacity. The CE of the pristine cell is slightly larger than that of the SnO_2_-CNT cell. This is because of the longer charge–discharge process and the larger charge loss in the SnO_2_-CNT cell. The voltage efficiency (VE) reveals the electrochemical activity of the electrode. The VE of the modified cell is 8.1% larger than the pristine cell (63.5%) at 150 mA cm^−2^. The energy efficiency (EE) depends on both the CE and VE. Both the EE and VE can be used to evaluate the electrochemical polarization of electrodes. The EE attenuation phenomenon occurs in both cells as the current density increases, but the cell with the SnO_2_-CNTs has a smaller attenuation than the pristine cell. The EE of the pristine and modified cells are 62.8% and 7% at 150 mA cm^−2^, respectively. These results demonstrate that the composite can reduce electrochemical polarization and improve electrochemical activity. Figures 5C,D show the charge–discharge curves of the pristine and SnO_2_-CNTs modified cells, respectively. The modified cell has a higher discharge voltage platform and a lower charge voltage platform, illustrating that the SnO_2_-CNTs composite can decrease electrochemical polarization and increase the energy density of the cell.

## Conclusion

In this study, the SnO_2_-CNT catalyst was prepared using a sol-gel method. The effects of the catalyst on the redox reaction and the cell performance were investigated. Compared with pure SnO_2_ and pure CNTs, the SnO_2_-CNTs show better electrocatalytic activity and reversibility for the ${\text{VO}}_{2}^{+}$/VO^2+^ and V^2+^/V^3+^ reactions. This is due to the synergistic catalysis of SnO_2_ and the CNTs. SnO_2_ mainly provides the catalytic active sites while the CNTs mainly provides the 3D structure and high electrical conductivity. Therefore, this composite has larger specific surface area and excellent synergistic catalytic performance. At current densities of 50–150 mA cm^−2^, the discharge capacity of the modified cell is higher than the pristine cell, indicating that a modified cell has a higher electrolyte utilization rate and better electrochemical stability. The EE of the modified cell (7%) is 7.2% higher than the pristine cell (62.8%) at 150 mA cm^−2^. The results show that this composite can provide significant benefits for the improvement of the VRFB performance.

## Data Availability Statement

The original contributions presented in the study are included in the article/supplementary material, further inquiries can be directed to the corresponding author/s.

## Author Contributions

XF was mainly responsible for experimental operations and drafting paper. JX was mainly responsible for the collecting and processing experimental data. TZ was mainly responsible for collecting information. ZZ was mainly responsible for drafting paper. CH was mainly responsible for making important modifications to the manuscript. LD was mainly responsible for reviewing the final manuscript for publication. LW was mainly responsible for designing the experiment. ZH are mainly responsible for the paper guidance. All authors contributed to the article and approved the submitted version.

## Conflict of Interest

The authors declare that the research was conducted in the absence of any commercial or financial relationships that could be construed as a potential conflict of interest.

## References

[B1] Bayeh A. W. Lin G.-Y. Chang Y.-C. Kabtamu D. M. Chen G.-C. Chen H.-Y.. (2020). Oxygen-Vacancy-rich cubic CeO_2_ nanowires as catalysts for vanadium redox flow batteries. ACS Sust. Chem. Eng. 8, 16757–16765. 10.1021/acssuschemeng.0c03861

[B2] Cheng C. Huang Z. Zhang R. Zhou J. Liu Z. Zhong H.. (2020). Synthesis of an emerging morpholine-typed Gemini surfactant and its application in reverse flotation carnallite ore for production of potash fertilizer at low temperature. J. Cleaner Product. 261:121121. 10.1016/j.jclepro.202.121121

[B3] Cheng D. Li Y. Zhang J. Tian M. Wang B. He Z.. (2020). Recent advances in electrospun carbon fiber electrode for vanadium redox flow battery: properties, structures, and perspectives. Carbon 170, 527–542. 10.1016/j.carbon.202.08.058

[B4] Cheng Y. Huang J. Qi H. Cao L. Yang J. Xi Q.. (2017). Adjusting the chemical bonding of SnO_2_@CNT composite for enhanced conversion reaction kinetics. Small 13:1700656. 10.1002/smll.201700656 28640435

[B5] Chuanchang L. Bo Z. Qingxia L. (2020). N-eicosane/expanded graphite as composite phase change materials for electro-driven thermal energy storage. J. Energy Storage 29:101339. 10.1016/j.est.202.101339

[B6] Etesami M. Abouzari-Lotf E. Ripin A. Mahmoud Nasef M. Ting T. M. Saharkhiz A.. (2018). Phosphonated graphene oxide with high electrocatalytic performance for vanadium redox flow battery. Int. J. Hydrogen Energy 43, 189–197. 10.1016/j.ijhydene.2017.11.050

[B7] Fetyan A. El-Nagar G. A. Derr I. Kubella P. Dau H. Roth C. (2018). A neodymium oxide nanoparticle-doped carbon felt as promising electrode for vanadium redox flow batteries. Electrochim. Acta 268, 59–65. 10.1016/j.electacta.2018.02.104

[B8] He G. Yan G. Song Y. Wang L. (2020). Biomass juncus derived nitrogen-doped porous carbon materials for supercapacitor and oxygen reduction reaction. Front. Chem. 8:226. 10.3389/fchem.202.00226 32351930 PMC7174754

[B9] He Z. Cheng G. Jiang Y. Li Y. Zhu J. Meng W.. (2020). Novel 2D porous carbon nanosheet derived from biomass: ultrahigh porosity and excellent performances toward V^2+^/V^3+^ redox reaction for vanadium redox flow battery. Int. J. Hydrog. Energy 45, 3959–3397. 10.1016/j.ijhydene.2019.12.045

[B10] Huang Z. Zhang S. Cheng C. Wang H. Liu R. Hu Y.. (2020a). Recycling lepidolite from tantalum–niobium mine tailings by a combined magnetic–flotation process using a novel gemini surfactant: from tailings dams to the “bling” raw material of lithium. ACS Sust. Chem. Eng. 8, 18206–18214. 10.1021/acssuschemeng.0c06609

[B11] Huang Z. Zhang S. Wang H. Liu R. Cheng C. Liu Z.. (2020b). “Umbrella” structure trisiloxane surfactant: synthesis and application for reverse flotation of phosphorite ore in phosphate fertilizer production. Jo. Agricult. Food Chem. 68, 11114–11112. 10.1021/acs.jafc.0c04759 32936618

[B12] Jiang Y. Cheng G. Li Y. He Z. Zhu J. Meng W.. (2020). Superior electrocatalytic performance of porous, graphitic, and oxygen-functionalized carbon nanofiber as bifunctional electrode for vanadium redox flow battery. Appl. Surf. Sci. 525:146453. 10.1016/j.apsusc.202.146453

[B13] Jiang Y. Cheng G. Li Y. He Z. Zhu J. Meng W.. (2021a). Promoting vanadium redox flow battery performance by ultra-uniform ZrO_2_@C from metal-organic framework. Chem. Eng. J. 415:129014. 10.1016/j.cej.2021.129014

[B14] Jiang Y. Du M. Cheng G. Gao P. Dong T. Zhou J.. (2021b). Nanostructured N-doped carbon materials derived from expandable biomass with superior electrocatalytic performance towards V^2+^/V^3+^ redox reaction for vanadium redox flow battery. J. Energy Chem. 59, 706–714. 10.1016/j.jechem.202.12.013

[B15] Kou Z. Lu Y. Miao C. Li J. Xiao W. (2020). High-performance sandwiched hybrid solid electrolytes by coating polymer layers for all-solid-state lithium-ion batteries. Rare Metals. 10.1007/s12598-020-01678-w. [Epub ahead of print].

[B16] Liu J. Yuan L. Yuan K. Li Z. Hao Z. Xiang J.. (2016). SnO_2_ as a high-efficiency polysulfide trap in lithium-sulfur batteries. Nanoscale 8, 13638–13645. 10.1039/c6nr02345b 27364768

[B17] Liu N. Li B. He Z. Dai L. Wang H. Wang L. (2021). Recent advances and perspectives on vanadium- and manganese-based cathode materials for aqueous zinc ion batteries. J. Energy Chem. 59, 134–159. 10.1016/j.jechem.202.1.044

[B18] Lv Y. Han C. Zhu Y. Zhang T. Yao S. He Z.. (2021). Recent advances in metals and metal oxides as catalysts for vanadium redox flow battery: properties, structures, and perspectives. J. Mater. Sci. Technol. 75, 96–109. 10.1016/j.jmst.202.09.042

[B19] Ma Q. Deng Q. Sheng H. Ling W. Wang H.-R. Jiao H.-W.. (2018). High electro-catalytic graphite felt/MnO_2_ composite electrodes for vanadium redox flow batteries. Sci. China Chem. 61, 732–738. 10.1007/s11426-017-9235-6

[B20] Mehboob S. Ali G. Shin H.-J. Hwang J. Abbas S. Chung K. Y.. (2018). Enhancing the performance of all-vanadium redox flow batteries by decorating carbon felt electrodes with SnO_2_ nanoparticles. Appl. Energy 229, 910–921. 10.1016/j.apenergy.2018.08.047

[B21] Nie Y. Xiao W. Miao C. Wang J. Tan Y. Xu M.. (2021). Improving the structural stability of Ni-rich LiNi_.81_Co_.15_Al_.04_O_2_ cathode materials with optimal content of trivalent Al ions doping for lithium ions batteries. Ceramics International 47, 9717–9726. 10.1016/j.ceramint.202.12.111

[B22] Park M. Jung Y. J. Kim J. Lee H. Cho J. (2013). Synergistic effect of carbon nanofiber/nanotube composite catalyst on carbon felt electrode for high-performance all-vanadium redox flow battery. Nano Lett 13, 4833–4839. 10.1021/nl402566s 24024628

[B23] Qiu H. Zhao L. Asif M. Huang X. Tang T. Li W.. (2020). SnO_2_ nanoparticles anchored on carbon foam as a freestanding anode for high performance potassium-ion batteries. Energy Environ. Sci. 13, 571–578. 10.1039/c9ee03682b

[B24] Tang J. Yang J. Zhou X. Yao H. Zhou L. (2015). A porous graphene/carbon nanowire hybrid with embedded SnO_2_ nanocrystals for high performance lithium ion storage. J. Mate. Chem. A 3, 23844–23851. 10.1039/c5ta06859b

[B25] Tian Q. Chen Y. Zhang W. Sui Z. Yang L. (2020). Reducing the excessive interior space of SnO_2_@C nanotubes by encapsulating SnO_2_ nanowires for high lithium storage. J. Alloys Compounds 820:153404. 10.1016/j.jallcom.2019.153404

[B26] Wang B. Jiang T. Hu H. Wu M. (2007). Investigation of Ir-modified carbon felt as the positive electrode of an all-vanadium redox flow battery. Electrochimica Acta 52, 6755–6762. 10.1016/j.electacta.2007.04.121

[B27] Wang D. Jiang S. Duan C. Mao J. Dong Y. Dong K.. (2020). Spinel-structured high entropy oxide (FeCoNiCrMn)_3_O_4_ as anode towards superior lithium storage performance. J. Alloys Compounds 844:156158. 10.1016/j.jallcom.202.156158

[B28] Wang T. Li C. Xie X. Lu B. He Z. Liang S.. (2020). Anode materials for aqueous zinc ion batteries: mechanisms, properties, and perspectives. ACS Nano 14, 16321–16347. 10.1021/acsnano.0c07041 33314908

[B29] Wang Z. Dong K. Wang D. Luo S. Liu X. Liu Y.. (2020). Constructing N-Doped porous carbon confined FeSb alloy nanocomposite with Fe-N-C coordination as a universal anode for advanced Na/K-ion batteries. Chem. Eng. J. 384:123327. 10.1016/j.cej.2019.123327

[B30] Wang Z.-Y. Jiang S.-D. Duan C.-Q. Wang D. Luo S.-H. Liu Y.-G. (2020). In situ synthesis of Co_3_O_4_ nanoparticles confined in 3D nitrogen-doped porous carbon as an efficient bifunctional oxygen electrocatalyst. Rare Metals 39, 1383–1394. 10.1007/s12598-020-01581-4

[B31] Wei L. Zhao T. S. Zeng L. Zhou X. L. Zeng Y. K. (2016). Copper nanoparticle-deposited graphite felt electrodes for all vanadium redox flow batteries. Appl. Energy 180, 386–391. 10.1016/j.apenergy.2016.07.134

[B32] Wu L. Shen Y. Yu L. Xi J. Qiu X. (2016). Boosting vanadium flow battery performance by Nitrogen-doped carbon nanospheres electrocatalyst. Nano Energy 28, 19–28. 10.1016/j.nanoen.2016.08.025

[B33] Wu X. Xu H. Lu L. Zhao H. Fu J. Shen Y.. (2014). PbO_2_-modified graphite felt as the positive electrode for an all-vanadium redox flow battery. J. Power Sour. 250, 274–278. 10.1016/j.jpowsour.2013.11.02124279888

[B34] Yang Z. Shancheng W. Jinqing P. Yutong T. Chuanchang L. Freddy Yin Chiang B.. (2020). Liquid thermo-responsive smart window derived from hydrogel. Joule 4, 2458–2474. 10.1016/j.joule.202.09.001

[B35] Ye J. Zhao X. Ma Y. Su J. Xiang C. Zhao K.. (2020). Hybrid membranes dispersed with superhydrophilic TiO_2_ nanotubes toward ultra-stable and high-performance vanadium redox flow batteries. Adv. Energy Mater. 10:1904041. 10.1002/aenm.201904041

[B36] Yu L. Lin F. Xiao W. Xu L. Xi J. (2019). Achieving efficient and inexpensive vanadium flow battery by combining CexZr_1−*x*_O_2_ electrocatalyst and hydrocarbon membrane. Chem. Eng. J. 356, 622–631. 10.1016/j.cej.2018.09.069

[B37] Zhang F. Teng X. Shi W. Song Y. Zhang J. Wang X.. (2020). SnO_2_ nanoflower arrays on an amorphous buffer layer as binder-free electrodes for flexible lithium-ion batteries. Appl. Surface Sci. 527:14691. 10.1016/j.apsusc.202.146910

[B38] Zhou X. Zhang X. Mo L. Zhou X. Wu Q. (2020). Densely populated bismuth nanosphere semi-embedded carbon felt for ultrahigh-rate and stable vanadium redox flow batteries. Small 16:1907333. 10.1002/smll.201907333 32789972

